# Linking cytoarchitecture to metabolism: sarcolemma-associated plectin affects glucose uptake by destabilizing microtubule networks in *mdx* myofibers

**DOI:** 10.1186/2044-5040-3-14

**Published:** 2013-06-12

**Authors:** Marianne Raith, Rocio G Valencia, Irmgard Fischer, Michael Orthofer, Josef M Penninger, Simone Spuler, Günther A Rezniczek, Gerhard Wiche

**Affiliations:** 1 Department of Biochemistry and Cell Biology, Max F. Perutz Laboratories, University of Vienna, Dr.-Bohr-Gasse 9, Vienna, 1030, Austria; 2 Institute of Molecular Biotechnology of the Austrian Academy of Sciences, Dr.-Bohr-Gasse 3, Vienna, 1030, Austria; 3 Muscle Research Unit, Experimental and Clinical Research Center, Lindenberger Weg 80, Berlin, 13125, Germany; 4 Department of Obstetrics and Gynecology (Marienhospital Herne), Ruhr-Universität Bochum, Düngelstrasse 33, Herne, 44623, Germany

**Keywords:** Plectin, Dystrophin, Sarcolemmal integrity, Glucose metabolism, Microtubules

## Abstract

**Background:**

Duchenne muscular dystrophy (DMD) is one of the most frequent forms of muscular disorders. It is caused by the absence of dystrophin, a core component of the sarcolemma-associated junctional complex that links the cytoskeleton to the extracellular matrix. We showed previously that plectin 1f (P1f), one of the major muscle-expressed isoforms of the cytoskeletal linker protein plectin, accumulates at the sarcolemma of DMD patients as well as of *mdx* mice, a widely studied animal model for DMD.

Based on plectin’s dual role as structural protein and scaffolding platform for signaling molecules, we speculated that the dystrophic phenotype observed after loss of dystrophin was caused, at least to some extent, by excess plectin. Thus, we hypothesized that elimination of plectin expression in *mdx* skeletal muscle, while probably resulting in an overall more severe phenotype, may lead to a partial phenotype rescue. In particular, we wanted to assess whether excess sarcolemmal plectin contributes to the dysregulation of sugar metabolism in *mdx* myofibers.

**Methods:**

We generated plectin/dystrophin double deficient (dKO) mice by breeding *mdx* with conditional striated muscle-restricted plectin knockout (cKO) mice. The phenotype of these mice was comparatively analyzed with that of *mdx*, cKO, and wild-type mice, focusing on structural integrity and dysregulation of glucose metabolism.

**Results:**

We show that the accumulation of plectin at the sarcolemma of *mdx* muscle fibers hardly compensated for their loss of structural integrity. Instead, it led to an additional metabolic deficit by impairing glucose uptake. While dKO mice suffered from an overall more severe form of muscular dystrophy compared to *mdx* or plectin-deficient mice, sarcolemmal integrity as well as glucose uptake of their myofibers were restored to normal levels upon ablation of plectin. Furthermore, microtubule (MT) networks in intact dKO myofibers, including subsarcolemmal areas, were found to be more robust than those in *mdx* mice. Finally, myotubes differentiated from P1f-overexpressing myoblasts showed an impairment of glucose transporter 4 translocation and a destabilization of MT networks.

**Conclusions:**

Based on these results we propose that sarcolemma-associated plectin acts as an antagonist of MT network formation in myofibers, thereby hindering vesicle-mediated (MT-dependent) transport of glucose transporter 4. This novel role of plectin throws a bridge between extra-sarcomeric cytoarchitecture and metabolism of muscle fibers. Our study thus provides new insights into pathomechanisms of plectinopathies and muscular dystrophies in general.

## Background

Plectin is an important cytolinker protein that is responsible for the networking and anchorage of intermediate filaments (IFs) to organelles and junctional complexes. It is expressed as multiple isoforms with different short N-terminal sequences (generated by alternative 5′-splicing) that determine their differential cellular targeting [1,2]. Being bound to different cellular structures via their N-termini and recruiting IFs via their C-termini, plectin isoforms play pivotal roles in shaping the cytoarchitecture of cells, with consequences for vital cellular features such as polarity and migratory potential [3,4]. Dysfunction or absence of plectin leads to epidermolysis bullosa simplex (EBS), a skin blistering disease that in most cases is associated with muscular dystrophy [5,6]. In myofibers, the contractile apparatus is suspended in a network of desmin filaments linked to the nuclear envelope and the sarcoplasmatic reticulum via plectin isoform 1 (P1), to sarcolemmal costameres and Z-disks via P1f and P1d, respectively, and to mitochondria via P1b [7-9]. Apart from functioning as structural reinforcement and organizing elements of the cytoskeleton, plectin isoforms play also an important role as scaffolding platforms for signaling proteins involved in cell metabolism, stress response, and motility [10-13].

Isoform P1f was found to be overexpressed at the sarcolemma of patients suffering from various types of muscular dystrophy as well as in regenerated muscle fibers of *mdx* mice, a mouse model for DMD [7,14]. X-chromosome-linked DMD is the most frequent form of muscular dystrophy with an incidence rate of 1 in 3,500 live male births [15]. It is characterized by the absence of full-length dystrophin, an approximately 400 kDa protein that is important for maintaining muscle fiber architecture [16]. Overexpression of plectin at the sarcolemma of DMD and *mdx* myofibers in response to the loss of dystrophin could be a compensatory mechanism for stabilizing costameres and the plasma membrane-associated protein skeleton. In fact, plectin was found to interact with the same binding domain of β-dystroglycan that normally is occupied by dystrophin [7].

Generally, *mdx* mice show a milder form of muscular dystrophy than DMD patients and in comparison live longer (for review see [17]). A larger muscle stem cell pool owing to the longer telomers of mouse chromosomes [18] and/or impaired differentiation of muscle fiber precursor (satellite) cells [19] has been suggested as being responsible for these differences. *Mdx* mice were also reported to have an increased body weight [20], in line with the impaired metabolism described for *mdx*-derived skeletal muscles [21] and dystrophin-deficient myoblasts [22]. Thus, the *mdx* mouse system has the potential to serve as a genetically manipulable tool not only for studying plectin’s role as structural reinforcement element of the sarcolemma but also for investigating plectin-dependent metabolic processes.

Thus, we hypothesized that the elimination of plectin expression in *mdx* skeletal muscle, while probably resulting in an overall more severe phenotype, may lead to a partial phenotype rescue. Especially, an interference of excess P1f with signaling and/or regulatory processes involved in glucose metabolism seemed conceivable. Glucose metabolism involves the translocation of glucose transporter 4 (GLUT4) from the cytoplasm to the sarcolemma as a result of insulin dependent or independent signaling pathways (for review see [23]). This translocation requires an intact cell membrane and cortical actin system to allow fusion of GLUT4-loaded vesicles with the sarcolemma, as well as a properly functioning microtubule (MT) network for long-distance vesicle transport (for review see [24]).

To test our hypothesis we investigated whether plectin indeed plays a role in glucose metabolism. For this, we crossed *mdx* mice with striated muscle-restricted conditional plectin knockout mice, thus generating a mouse line that in addition to dystrophin was lacking plectin in myofibers. We show that the ablation of plectin, while drastically reducing the lifespan and worsening the overall phenotype of *mdx* mice, led to a reversion of impaired glucose uptake and partial restoration of sarcolemmal integrity in their muscle fibers. On the mechanistic level, we show that sarcolemma-associated plectin acts as a destabilizer of MTs and thereby affects the translocation of GLUT4.

## Methods

### cDNA constructs

Full-length mouse P1f-EGFP (pGR258) has been described previously [2]. pmCherry-HA-GLUT4 is an expression plasmid that encodes mCherry-tagged human GLUT4 containing a hemagglutinin (HA) tag within an exofacial loop, generated by inserting a BamHI/HindIII fragment from GFP-HA-GLUT4 (a kind gift of Samuel W Cushman, NIDDK, Bethesda, MD, USA [25]) into the corresponding sites of a modified pmCherry-C1 (a kind gift of Anna Akhmanova, Erasmus MC, Rotterdam, Netherlands, who originally received mCherry cDNA from R Y Tsien, University of California, San Diego, CA, USA); a shift of the open reading frame by two bases was introduced by BglII-digestion, incubation with mung bean nuclease, and subsequent religation. mCherry-HA was generated by inserting an oligonucleotide (HA fw: 5′-GAT CTT ACC CAT ACG ATG TTC CAG ATT ACG CTT GAG GTA C-3′; HA rev: 5′-CTC AAG CGT AAT CTG GAA CAT CGT ATG GGT AA-5′) encoding the HA-tag after annealing and restriction with BglII/KpnI into the corresponding sites of pmCherry-C1.

### Mice and cell lines

Experiments involving mice were approved by the Austrian Federal Government. Conditional striated muscle-restricted plectin knockout (cKO) mice lacking all isoforms of plectin were generated by breeding plectin floxed mice with muscle creatine kinase (MCK)-Cre mice (provided by C R Kahn, Joslin Diabetes Center and Harvard Medical School, Boston, MA, USA) as previously described [8]; for *mdx* mice see [26]. dKO mice were generated by breeding cKO with *mdx* mice. Unless otherwise stated, eight to ten-week-old male littermates were used for experiments. Primary myoblasts were isolated from two to three-day-old wt, *mdx*, cKO, or dKO newborns following established protocols [27]. After two to three passages, cells were differentiated to myofibers for seven days. For some experiments (double transfection with P1f/GLUT4, and counting of MTs) an immortalized mouse myoblast cell line was used [11].

### Antibodies

For immunofluorescence microscopy (IFM) and immunoblotting (IB) the following antibodies were used: mAb to α-tubulin (IFM; Acris, Herford, Germany), mAb to desmin (IFM; Dako Cytomation, Glostrup, Denmark), antisera #9 (IB) and #46 (IFM) to plectin [28], anti-GLUT4 (IFM; a kind gift from Dr. Hadi Al-Hasani, German Diabetes Center, Düsseldorf, Germany; IB; #2299; Cell Signaling, Danvers, MA, USA), anti-sarcomeric α-actinin (IFM; Sigma, St. Louis, MO, USA), anti-dystrophin (IB; NCL-Dys1; Leica Biosystems Newcastle Ltd, Newcastle upon Tyne, UK), anti-α-tubulin (IB; DM1A, Abcam, Cambridge, UK), anti-acetylated tubulin (IB; 6-11-B1; Sigma, St. Louis, MO, USA), anti-tau (IB; A0028; Dako, Glostrup, Denmark), and mAB to HA-tag (GLUT4 translocation assay; HA.11; Covance, Berkeley, CA, USA). As secondary antibodies we used donkey anti-rat 633, goat anti-rat Cy5, goat anti-mouse 488, and donkey anti-mouse Dylight 649 for IFM and HRPO-conjugated goat anti-rabbit or goat anti-mouse for IB (all Jackson ImmunoResearch, West Grove, PA, USA).

### Histology

Histological analyses were performed using cryosections following standard protocols. Fiber types were assigned based on ATPase (pH 4.2) staining. To measure fiber diameters, individual fibers were manually circumscribed with polygons and, in order to compensate for skewed sections, a custom computer program then calculated diameters as the maximal length inside each polygon orthogonal to the largest diameter of the polygon.

### Immunohistochemistry

Teased muscle fibers were prepared in MT-stabilizing buffer from EDL muscle as described previously [7] and MTs were stained with antibodies to α-tubulin and 633-conjugated secondary antibodies. Myofibers derived from myoblasts co-transfected with pmCherry-HA-hGLUT4 and pGR258 (plectin 1f-EGFP) were immunolabeled using mAbs to α-tubulin and goat anti-rat Cy5-conjugated secondary antibodies after fixing the cells in 4% PFA at room temperature. To measure MT stability in primary myoblasts, cells were incubated with 1 μM nocodazole for 30 minutes at 37°C, washed, fixed for 30 minutes with 2.5% PFA, and stained with mAbs to α-tubulin/Cy5 and mAbs to desmin/488. The total length of MTs was then measured in randomly chosen microscopic fields using LSM510 software (Zeiss, Oberkochen, Germany) in an observer-blinded manner and divided by the area occupied by cells. Nuclei were stained using Hoechst 33258 (Sigma, St. Louis, MO, USA).

GLUT4-specific signals in peripheral (sarcolemmal) and interior (cytoplasmic) subcompartments of cryosectioned QF muscle fibers were quantified by manually inscribing and circumscribing the sarcolemmal regions of individual fibers with polygons and measuring the fluorescence intensity per unit area in the two resulting compartments using ImageJ software (NIH Image, Bethesda, MD, USA).

### Immunoblotting

Protein expression levels were determined densitometrically after separation of proteins contained in GC muscle lysates by SDS-PAGE, subsequent transfer to nitrocellulose and immunodetection using antisera to plectin, dystrophin, GLUT4, α-tubulin, acetylated tubulin, or tau; and HRPO-conjugated goat anti-rabbit or goat anti-mouse secondary antibodies. Quantification of bands was performed using Quantiscan (Biosoft, Cambridge, UK). Expression levels were normalized to total protein content which was determined from corresponding Coomassie-stained gels by measuring the intensities of four different bands per lane that showed the same relative intra-lane intensities.

### Oral glucose tolerance test (oGTT) and insulin measurement

oGTT was performed after fasting mice overnight. Mice were force-fed by an oral gavage with glucose (2 g/kg body weight) and blood samples were collected from the tail vein at the indicated time points. Blood glucose and insulin levels were determined with a standard glucometer (OneTouch Ultra 2, Lifescan, Milpitas, CA, USA) and a low sample volume insulin ELISA (Mercodia, Uppsala, Sweden), respectively.

### Differential blood count and CK measurements

Blood smears were stained with May-Gruenwald-Giemsa solution and one hundred white blood cells were counted per slide. CK activity in the plasma was determined using CK-NAC FS reagent (DiaSys, Holzheim, Germany) according to the manufacturer’s instructions. Additionally, CK activity was analyzed in diluted total muscle lysates of GC muscle using a similar protocol. CK mRNA expression was measured by real-time RT-PCR on cDNA samples transcribed from 1 μg total RNA prepared from QF muscle using as primers: 5′-CCT GTT TGA TCC CAT CAT CC-3′ (fw) and 5′-AGC ACA TAG TTG GGG TCC AG-3′ (rev). For normalization, primers for HPRT1 (fw: 5′-CAG GCC AGA CTT TGT TGG AT-3′; rev: 5′-TTG CGC TCA TCT TAG GCT TT-3′) were used.

### EBD penetration assay

Mice were injected intraperitoneally with 10 μls/g body weight of a sterile 1% EBD solution in PBS, and after 24 hours sacrificed by cervical dislocation. Dissected extensor digitorum longus (EDL) muscles were immediately transferred to isopentane pre-cooled in liquid nitrogen. Five-μm-cross-sections were prepared using a cryotome (Zeiss HM 500 OM, Zeiss, Oberkochen, Germany) and stored at -80°C until use. To check for EBD-positive fibers, cryosections were fixed for one minute with acetone, mounted in Histofluid, and subjected to immunofluorescence analysis using a bandpass filter (575 to 640 nm). Whole cross-sectional areas of two cryosections from three different animals per genotype were scored for EBD positive fibers.

### GLUT4 translocation assay

Mouse myoblasts were transfected with pmCherry-HA-hGLUT4 and pGR258 (P1f-EGFP) or pmCherry-HA using the Amaxa NHDF nucleofector kit (Lonza, Cologne, Germany) according to the manufacturer’s instructions (program U-023) and differentiated for seven days. Differentiated myoblasts were washed twice with PBS and incubated for 10 minutes in Krebs Ringer bicarbonate solution (KRB). Cells were then incubated in KRB containing 100 μU insulin (Eli Lilly, Toronto, Canada) and 120 μg anti-HA-tag antibodies for 20 minutes at 37°C. After two washing steps with KRB, cells were incubated with secondary antibody (Dylight 649) diluted (2.8 μg/ml) in KRB. Cells were fixed for 90 seconds with ice-cold methanol, washed twice with PBS and once with water, and mounted in Mowiol 4–88 (Hoechst, Frankfurt, Germany). Digital images were obtained using a Zeiss LSM 510 confocal microscope (Carl Zeiss, Oberkochen, Germany) and colocalization was measured in ImageJ (http://rsbweb.nih.gov/ij/) using Costes’ approach [29].

### Statistical analyses

Kruskal-Wallis and Mann–Whitney *U* tests were used for comparison of the four different genotypes tested. Student’s *t*-test was used for evaluation of GLUT4 translocation, MT counting, and MT stability in primary myofibers. Calculations were done in SPSS 16.0 (IBM, Armonk, NY, USA).

## Results

### Plectin deficiency aggravates the muscular dystrophy phenotype of *mdx* mice

**Figure 1:**
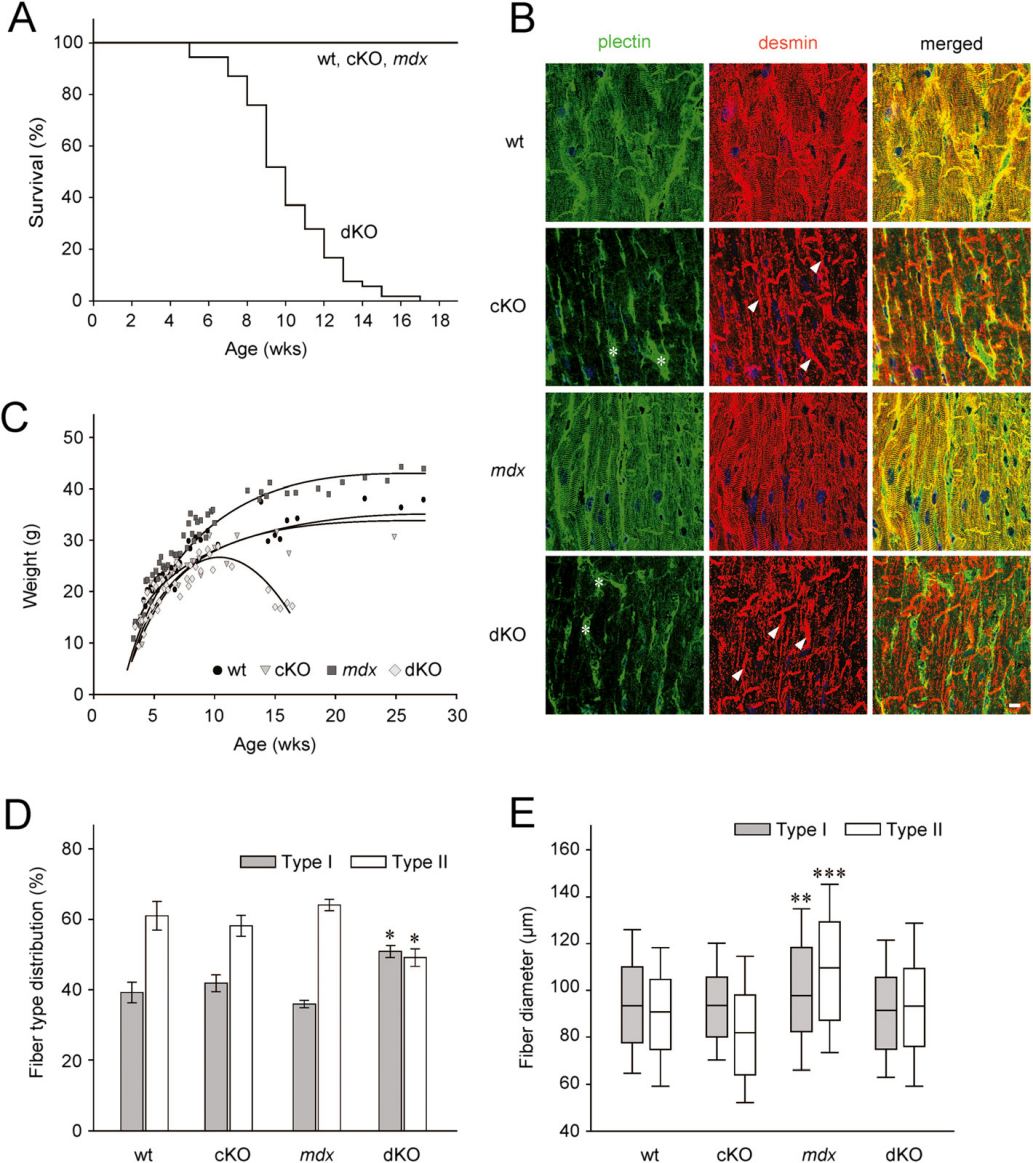
**Phenotypic analyses of dKO mice compared to wt, cKO, and *mdx *mice. (A)** Lifespan (n = 54 per genotype). **(B)** Immunofluorescence microscopy of heart cryosections. Note aggregates of desmin (arrows) and plectin-positive connective tissue (asterisks) in cKO and dKO samples. Bar, 10 μm. **(C)** Body weight. Note reduced weight of dKO mice compared to wt and cKO littermates, and increased weight of *mdx* mice (n ≥ 55 per genotype). **(D)** Fiber type distribution in soleus muscle determined by ATPase staining (pH 4.2). Values represent percent mean ± SEM of total fibers (n ≥ 3 per genotype; *P < 0.05). **(E)** Diameters of type I and II fibers. Median (line in box), 25^th^ (bottom line of box), 75^th^ (top line of box), 5^th^ and 95^th^ (whiskers) percentiles are indicated (n ≥ 3 per genotype; **P < 0.01, ***P < 0.001).

To assess plectin’s contribution to the phenotype displayed by *mdx* mice, dystrophin/plectin double KO mice (dKO) were bred by crossing *mdx* with striated muscle-restricted conditional plectin knockout (cKO) mice. To allow for optimal comparison of phenotypes, a breeding scheme was employed that generated all four genotypes of interest (wt, MCK-Cre/cKO, *mdx*, and dKO) in the male offspring within single litters (plectin and dystrophin immunoblots are shown in Additional file 1: Figure S1). The lifespan of dKO mice (5 to 17 weeks) turned out to be considerably lower than that of cKO or *mdx* mice, which showed increased mortality rates only after 26 weeks (cKO; [8]), or an almost normal lifespan of approximately 21 months (*mdx*; [30]; and data not shown) (Figure 1A). As the MCK promoter which drives the Cre-mediated knockout of plectin in skeletal muscle is also active in heart, the shorter life span of dKO mice could have been due to heart failure. In fact, the desmin IF system was severely disturbed in dKO mice, as revealed by immunostaining of cryosectioned hearts (Figure 1B). However, the extent of IF disorganization and aggregate formation was very similar to that observed in the considerably longer-lived cKO mice [8]. For this reason we considered it unlikely that the lack of plectin in the hearts of dKO mice was the primary cause of their death. More likely, the shorter lifespan of dKO mice was due to their strong muscle wasting which was paralleled by a reduction of their body weight (peaking at around 10 weeks of age and rapidly declining thereafter; Figure 1C). In contrast, *mdx* mice showed an increased body weight, consistent with a reduced metabolism.

Comparative histopathological analyses of soleus and EDL muscles from 10-week-old dKO, cKO, *mdx*, and wt mice revealed atrophic fibers, fibers with central nuclei, and an increase in fibrotic tissue in dKO mice (Additional file 2: Table S1 and Additional file 3: Figure S2). Furthermore, aggregates of mitochondria were more prominent in dKO compared to cKO muscle [8] while no abnormalities in the distribution of mitochondria were observed in *mdx* muscle. Contrary to cKO and *mdx* fibers, the majority of dKO fibers appeared to be under high oxidative stress as indicated by strong signals for NADH. This was particularly striking in the case of EDL, a muscle predominantly consisting of glycolytic type II fibers which generally have a lower oxygen capacity (Additional file 3: Figure S2).

Using the adenosine triphosphatase (ATPase) reaction at pH 4.2 to analyze the fiber type composition of dKO soleus muscle, a statistically significant shift from fast-twitch glycolytic type II fibers to slow aerobic (oxidative) type I fibers was observed (Figure 1D). Furthermore, while in *mdx* muscles, compared to wt, a higher number of hypertrophic fibers and increased heterogeneity in the diameters of both type I and type II fibers were observed (see also [31]), in dKO mice the diameters of both fiber types were similar to those of wt littermates (Figure 1E). This observation would be consistent with the idea that plectin deficiency led to a rescue of the hypertrophy phenotype typical of *mdx* mice. However, since it is known that active muscle contraction is another important factor for skeletal muscle size and fiber type maintenance, the normal size of dKO myofibers could have equally well been due to the visually observable decreased physical activity of dKO mice.

The severe necrosis in dKO muscle was also reflected in differential blood cell counts showing an increased proportion of neutrophile granulocytes and a corresponding reduction of lymphocytes (Additional file 4: Figure S3). In summary, although the combined loss of plectin and dystrophin, as predicted, led to an aggravation of the dystrophic muscular phenotype, a number of abnormalities observed in *mdx* mice were fully or partially rescued after deletion of plectin (see also below).

### Plectin deficiency restores sarcolemmal integrity in *mdx* mice

**Figure 2:**
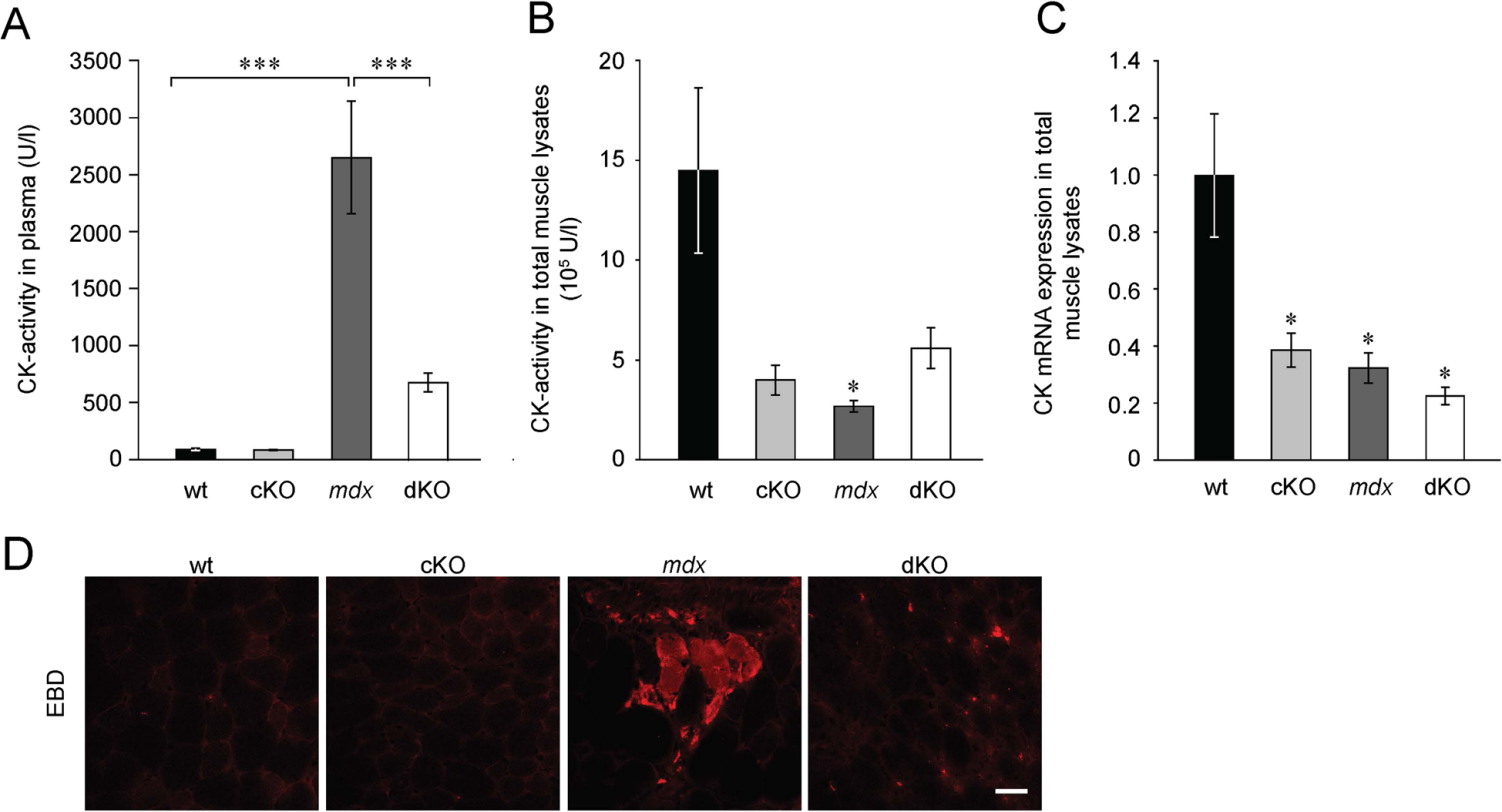
**Measurements of sarcolemma integrity. (A** and **B)** CK activities were determined in plasma **(A)** (n = 6 per genotype; ***P < 0.001) and in GC muscle tissue lysates **(B)** (n = 3 per genotype; *P < 0.05). **(C)** CK mRNA levels in QF muscle lysates were determined by qPCR relative to the levels of the reference gene HPRT1 and normalized to wt muscle (n = 4 per genotype; *P < 0.05). **(D)** EBD penetration into EDL muscle tissue was assessed by immunofluorescence microscopy of cryosections from dye-injected mice (n = 3 per genotype, whole cross-sectional areas of two cryosections per genotype were examined). Note EBD-positive fibers in *mdx* mice. Data in **A**-**C** are presented as mean ± SEM.

Plasma levels of creatine kinase (CK), which serve as an indicator for sarcolemma integrity [32], showed an approximately 30-fold increase in *mdx* over wt mice, whereas in dKO mice they were only approximately eight-fold increased (equivalent to an approximately four-fold lower CK levels in dKO compared to *mdx* mice). cKO mice showed non-pathological levels of CK (Figure 2A). When CK activities were measured in muscle lysates, the situation was different, as in this case all the mouse lines affected by muscular dystrophy, including cKO mice, showed reduced activities without any significant differences among them (Figure 2B). A similar pattern was observed for CK mRNA expression levels (Figure 2C). In an alternative test of sarcolemma integrity, the barrier function of myofibers was assessed after peritoneal injection of Evans Blue dye (EBD). While in *mdx* mice EBD-positive myofibers were clearly detectable, in wt, cKO, and dKO mice no dye penetration was observed (Figure 2D). These observations were consistent with the markedly increased plasma CK levels of *mdx* mice (Figure 2A).

### Metabolic defects of *mdx* mice are absent from dKO mice

**Figure 3:**
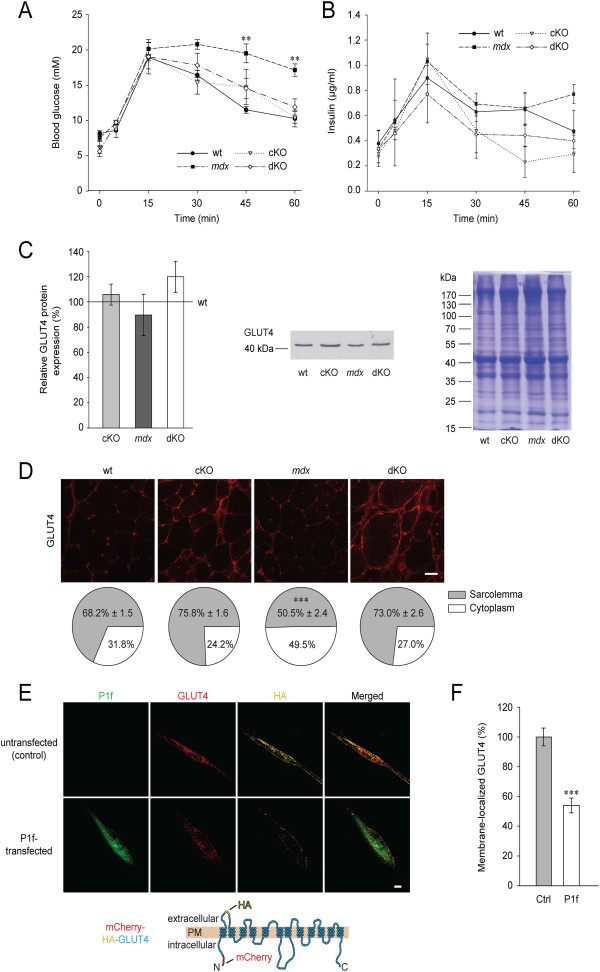
**Analysis of glucose metabolism and visualization of GLUT4 translocation. (A)** Blood glucose levels of wt and mutant mice were assessed by oGTT (n ≥ 5 per genotype). Note elevated glucose levels in *mdx* mice, 30, 45, and 60 minutes after force-feeding of glucose (**P < 0.01). **(B)** Insulin levels were measured in blood during oGTT (n ≥ 5 per genotype, differences are not statistically significant). **(C)** Bar graph, representative immunoblot, and corresponding Coomassie-stained gel, showing GLUT4 protein levels in mutant relative to wt (100%) GC muscles (n = 4 per genotype). Differences are not statistically significant. Positions of molecular mass markers are indicated. **(D)** Immunolocalization and quantification of GLUT4 on cryosections of QF muscles from wt and mutant mice. Note increased immunoreactivity of GLUT4 at the periphery of myofibers in wt, cKO and dKO compared to *mdx* mice (***P < 0.001); details of quantification are described in the text. **(E)** Fluorescence microscopy of GFP-P1f-overexpressing (P1f-transfected) and control (Ctrl; GFP-P1f-untransfected) differentiated myotubes, both expressing a mCherry-HA-GLUT4 fusion protein; a topographic scheme of the mCherry-HA-GLUT4 fusion protein is shown below micrographs. Note reduced labeling of HA (yellow) in P1f-transfected compared to control myotubes. Bar, 10 μm. **(F)** Quantification of GLUT4 translocation. GLUT4 (mCherry) signals colocalizing with extracellular HA-tag signals were measured as described in the text. Note, surface GLUT4 labeling was reduced to 54% in differentiated myotubes overexpressing GFP-tagged P1f (P1f) compared to control cells (Ctrl) (n ≥ 13 per genotype; four independent experiments, ***P < 0.001). Bar, 10 μm. Data in **(A-D)**, and **F** represent mean ± SEM.

The increased body weight of *mdx* mice ([20]; and Figure 1C) and the previously reported defects in metabolic regulation observed in *mdx* skeletal muscle [21] and dystrophin-deficient myoblasts [22], were consistent with a deregulation of sugar uptake. To directly assess whether plectin’s sarcolemmal accumulation in *mdx* muscle fibers contributed to such deregulation, wt and mutant mice were subjected to oral glucose tolerance tests (oGTT). Interestingly, while confirming that the uptake of blood sugar by *mdx* muscle was severely hampered (Figure 3A), these tests revealed normal glucose uptake in dKO as well as in cKO mice. Thus, plectin deficiency seemed to restore the glucose uptake capacity of *mdx* muscle, thereby rescuing its metabolic deficit. Measurements of plasma insulin levels showed that all types of mice responded normally to force-fed glucose (Figure 3B), demonstrating that insulin secretion was not affected. To investigate whether insulin-independent metabolic mechanisms had any effect on glucose uptake, we determined expression levels of the active (subunit alpha-phosphorylated) form of AMP-activated kinase (AMPK), a key player in insulin-independent signaling (for a review see [33]). However, no significant differences between wt and mutant mice were found (data not shown).

### Normal expression levels but compromised translocation of GLUT4 in *mdx* muscle

As the major insulin-dependent glucose transporter in muscle and adipose tissues, GLUT4 is largely responsible for the decline of blood glucose levels after food consumption. To examine whether the observed metabolic phenotype of *mdx* mice and its rescue in dKO mice were reflected in altered expression levels or intracellular localization of GLUT4, we quantitated total GLUT4 protein levels in muscle lysates by immunoblotting and measured the relative intensities of GLUT4-specific immunostaining in peripheral (sarcolemmal) and interior (cytoplasmic) subcompartments of cryosectioned QF muscle fibers. While immunoblots showed that the levels of GLUT4 were similar in all genotypes (Figure 3C), quantitative analysis of immunofluorescent images clearly revealed a higher concentration of GLUT4 in peripheral versus interior regions in wt, cKO, and dKO fibers, but not in those from *mdx* muscle (Figure 3D).

To establish a direct link between sarcolemmal-associated plectin and GLUT4 translocation, we developed an assay where GLUT4 translocation could be monitored *ex vivo*. For this, we mimicked the plectin-specific situation in *mdx* muscle fibers by overexpressing a GFP-tagged variant of the sarcolemma-associated plectin isoform P1f in a myoblast cell line [11] that expresses dystrophin at normal levels. To monitor GLUT4 and visualize translocated molecules at the same time, cells were cotransfected with an expression plasmid encoding mCherry-GLUT4 with an additional antibody-detectable HA-tag in its extracellular domain. After transfection, cells were subjected to differentiation for seven days and were then incubated with insulin to stimulate GLUT4 translocation. Scoring myofibers for membrane-recruited GLUT4 (co-localization of HA-tag and mCherry signals) in GFP-negative (control) and GFP-positive (P1f-overexpressing) myofibers (Figure 3E), we found GLUT4 translocation to the plasma membrane to be reduced by 46% (*P* < 0.001) in myofibers overexpressing P1f (Figure 3F). A number of control experiments supported the validity of these results. First, when myoblasts were transfected with a plasmid (mCherry-HA) encoding a fusion protein of mCherry and the HA-tag without the GLUT4 sequence, no extracellular HA immunoreactivity was detectable, whereas after fixation and permeabilization of cells, the HA-tag was clearly visualized (Additional file 5: Figure S4). Second, the protein levels of overexpressed P1f in cultured myotubes (estimated by quantitative immunofluorescence microscopy) were twice as high (approximately 200%) as those in non-transfected cells; thus they were in the range of the P1f levels estimated for *mdx* myofibers (approximately 170% total plectin, and approximately 150% P1f in membrane preparations; see [7], and data not shown). Third, testing the maturity of the myofibers used in the translocation assay, immunofluorescence microscopy revealed a pronounced striated staining pattern of sarcomeric α-actinin, typical of mature myofibers (Additional file 6: Figure S5). Together, the reduced GLUT4 translocation upon overexpression of P1f observed *ex vivo* and the decreased levels of sarcolemma-associated GLUT4 seen *in vivo*, provided strong evidence for sarcolemma-associated plectin directly affecting GLUT4-trafficking, albeit the underlying mechanism remained obscure.

### Plectin destabilizes subsarcolemmal MT networks

**Figure 4:**
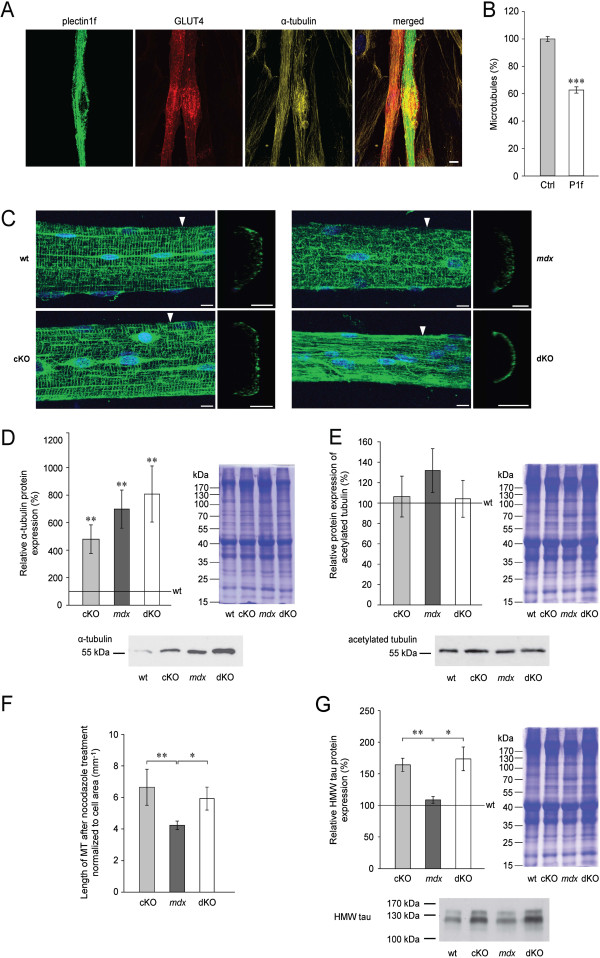
**Comparative phenotypic analyses of MT networks in wt and mutant muscles. (A)** Fluorescence microscopy imaging of P1f, GLUT4, and tubulin in GFP-P1f/mCherry-GLUT4-double transfected myocyte cultures after seven days of *in vitro* differentiation. Of the two adjacent cells shown in the center, one is double-transfected, overexpressing full-length P1f (green) and GLUT4 (red), while the other is single transfected, expressing only GLUT4. Note reduced density of MT network (amber) in the P1f-expressing cell (especially in regions with high P1f expression) due to lower number of MTs. Blue, nuclei. Bar, 10 μm. **(B)** Quantification of MTs in differentiated myocytes with or without overexpressed P1f. Note reduction of MT-specific signal in myotubes expressing GFP-tagged P1f (P1f) to 62.7% relative to control cells (Ctrl, 100%) (n ≥ 12 per genotype; four different myotubes per genotype; ***P < 0.001). **(C)** Teased EDL muscle fibers (from wt and mutant mice) were immunolabeled using primary antibodies to α-tubulin. Arrowheads, positions of virtual cross-sections shown in small panels. Bars, 10 μm. Blue, nuclei. **(D** and **E)** Quantitative immunoblotting analysis of GC muscle lysates using antibodies to α-tubulin **(D)** (n = 4 per genotype, **P < 0.01 compared to wt), or antibodies to acetylated tubulin **(E)** (n = 4 per genotype), with corresponding Coomassie-stained gels. Values represent percentages relative to wt (100%); are shown. Note that in **(E)** no significant differences were observed between any of the samples. **(F)** Total length of MTs (per cell area) measured after nocodazole treatment of primary myofibers. MTs were visualized as in **(C)**; (n ≥ 65 per genotype; *P < 0.05, **P < 0.01). Representative examples of cells subjected to the measurements are shown in Additional file 7: Figure S6. **(G)** Quantitative immunoblotting analysis of GC muscle lysates using antibodies to tau with corresponding Coomassie-stained gel. Note elevated levels of high molecular weight (HMW) isoforms of tau in dKO and cKO muscles compared to wt (100%) and *mdx* (109%) specimens (n = 4 per genotype; *P < 0.05, **P < 0.01). Positions of molecular mass marker (kDa) are indicated in immunoblots and corresponding Coomassie-stained gels **(D, E,** and **G)**. Data in bar graphs **(D-G)** are presented as mean ± SEM.

GLUT4 translocation occurs in the cytoplasm via storage vesicles that are transported along MTs to the cell periphery upon activation of the insulin receptor signaling pathway [34,35]. We reasoned that the compromised GLUT4 translocation observed in myocytes overexpressing P1f might be due to MT network alterations effected by plectin. To assess this idea, we first visualized MT networks of myofibers cotransfected with GFP-P1f and mCherry-GLUT4 by immunofluorescence microscopy using anti-α-tubulin antibodies and compared them with those of myofibers transfected with mCherry-GLUT4 alone (no GFP-P1f). As best seen in juxtaposed transfected and non-transfected cells (Figure 4A) and confirmed by quantitative analysis (Figure 4B), the MT network density was substantially reduced in P1f-overexpressing fibers (to approximately 63%). Furthermore, we observed that MTs were especially scarce in regions with high concentrations of P1f and more prominent in P1f-free regions, such as around the nuclei.

To visualize subsarcolemmal MT networks in intact myofibers, we prepared teased myofibers from EDL muscle of adult mice and subjected them to confocal immunofluorescence microscopy (Figure 4C). In specimens from *mdx* mice, MT networks were found to be reduced and disorganized (particularly in subsarcolemmal regions) compared to wt mice. Disorganization of MT networks in *mdx* mice, originally reported by Percival *et al*. [36], has recently been attributed to the loss of dystrophin’s MAP-like MT-stabilizing function [37]. In sharp contrast, in cKO and more so in dKO mice, MT networks were more prominent, appearing more robust compared to wt mice. Especially in fibers from dKO mice, thick, longitudinal bundles located underneath the sarcolemma were strikingly visible (Figure 4C). The differences in sarcolemma-associated MTs became particularly evident in virtual cross sections, where prominent tubulin-positive patches were clearly visible at the sarcolemma of wt, cKO, and dKO fibers, but were rarely seen in *mdx* fibers (Figure 4C, right panels). These data suggested that sarcolemma-associated dystrophin and plectin were influencing MT network formation at the plasma membrane in antagonistic ways. As MT networks in fibers without plectin (dKO and cKO) were more prominent than in wt fibers, while the opposite was true for *mdx* fibers (where plectin was overexpressed), plectin, contrary to dystrophin, seemed to destabilize MTs.

Tubulin incorporated into stably assembled MTs becomes acetylated by an acetyltransferase while disassembled tubulin as well as highly dynamic MTs remain unacetylated. This post-translational modification thus serves as a marker of MTs endowed with a long half-life. To gain insight into the dynamic state of MTs in myofibers of *mdx* versus cKO and dKO mice, we determined the protein expression levels of α-tubulin and the proportion of acetylated tubulin. Unexpectedly, we found that the levels of total (soluble and insoluble) α-tubulin were extensively (four to eight-fold) increased in muscles from mice with muscular dystrophy compared to wt littermates (Figure 4D). However, the amount of acetylated tubulin was not, or only insignificantly increased in cKO, *mdx*, and dKO muscles (Figure 4E), indicating that the bulk of tubulin found in these samples was unacetylated.

To assess and compare MT stability in mutant myocytes in a more direct way, we isolated primary myoblasts from cKO, *mdx*, and dKO littermates, let them differentiate to myotubes, and exposed these to low doses of the MT-depolymerizing drug nocodazole. By determining the total lengths of drug-resistant MTs per cell area by immunofluorescence microscopy, we found that MT polymers were clearly more abundant in cKO and dKO compared to *mdx* myotubes (Figure 4F, representative examples of measured cells are given in Additional file 7: Figure S6). This indicated that MTs in *mdx* myotubes were less stable than those in plectin-deficient cells. As the stability of MTs is regulated by MAPs, we determined the expression levels of tau, one of the main MAPs expressed in muscle tissue [38]. When cell lysates prepared from whole muscles were compared by immunoblotting, the levels of tau found in the cKO and dKO samples were significantly higher than in the *mdx* samples (Figure 4G) consistent with the data shown in Figures 4C and F. Similar observations were made for MAP4, another MAP expressed in muscle (data not shown). Based on these data it appears that sarcolemma-associated dystrophin and plectin have antagonistic impacts on the dynamics of subplasma membrane MT networks. While dystrophin stabilizes these networks, plectin destabilizes them. This mechanism would explain why the elimination of plectin from *mdx* muscle fibers rescues their capacity to recruit MTs to the membrane, thereby restoring GLUT4 translocation.

## Discussion

Having shown previously that P1f is overexpressed at the sarcolemma of *mdx* mice [7], in this study we asked the question whether plectin overexpression was contributing to the *mdx* muscle tissue necrosis phenotype, or whether it had an ameliorating effect. By comparing the histopathology of plectin/dystrophin dKO, *mdx,* and plectin cKO mice, it became clear that, overall, the additional lack of plectin in dKO mice was aggravating the muscular dystrophy phenotype of *mdx* mice, not at least because of the early death of double-deficient mice. The overexpression of plectin in *mdx* muscle could be seen as a cellular response to dystrophin deficiency that counteracts the compensatory action of upregulated utrophin under these conditions [39]. While our study demonstrates that plectin’s accumulation at the sarcolemma of regenerating *mdx* muscle fibers does not relieve their structural deficits but rather adds an additional deficit that affects the metabolism of the fiber by inhibiting its glucose uptake, pharmacologically-induced further upregulation of utrophin was shown to ameliorate the dystrophic phenotype of *mdx* muscle [40]. Our study further suggests that the reduced glucose metabolism of *mdx* mice is due to excessive subsarcolemmal plectin acting as a local antagonist of MT network formation in peripheral areas of myofibers with severe consequences for MT-dependent functions.

The molecular mechanism underlying MT destabilization through excess plectin in myofibers has yet to be unraveled. It is possible that plectin affects MTs either directly by inhibiting tubulin assembly into polymers, or indirectly by acting as a deregulator of MT assembly-promoting MAPs. We consider deregulation of MAPs as the more likely mechanism in light of plectin’s known interaction with various MAPs [41,42], including the tau isoforms shown to be expressed in skeletal muscle (Figure 4G). Moreover, a similar destabilizing effect of plectin on MTs could recently be demonstrated in keratinocytes [42].

**Figure 5:**
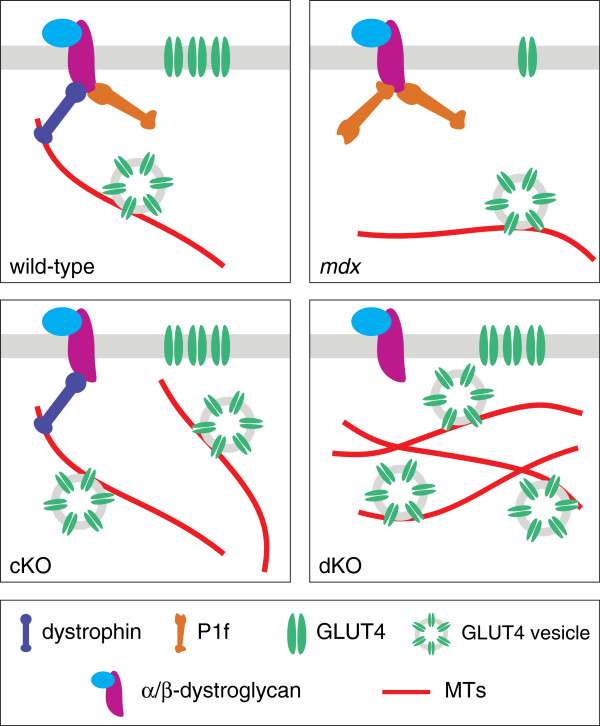
**Schematic model of GLUT4 and MT distribution in wt, *mdx*, cKO, and dKO myofibers.** Plasma membrane resident GLUT4 and juxtamembrane-positioned MTs are depicted as being reduced in (P1f*-*overexpressing) *mdx* myofibers compared to the other genotypes; whereas in cKO and dKO myofibers subsarcolemmal MT network densities are increased. The model makes differences in MT network density and membrane anchorage accountable for how efficiently GLUT4-containing vesicles are transported towards the sarcolemma, with a low point in the case of *mdx* muscle.

As proposed in the model presented in Figure 5, incorporation of GLUT4 into the membrane is reduced in P1f-overexpressing *mdx* myofibers (consistent with their defective glucose uptake), whereas under normal conditions (wild-type), or in situations where no plectin is encountered at the membrane (cKO, dKO; and where glucose uptake is normal), insulin-stimulated GLUT4 transport towards the membrane can take place along MTs in an undisturbed way. We expect that plectin affects also other types of MT-dependent vesicular transport processes, one of which could be the transport of dysferlin. As a protein involved in the secondary (inflammatory) response to injury [43-45], it has been suggested that dysferlin is translocated along MTs due to its *in vitro* interaction with α-tubulin and partial colocalization with polymerized MTs [46]. In this context it is of interest that dysferlin expression levels in GC muscle cell lysates from dKO mice were found to be two- to three-fold increased, compared to cKO and *mdx* mice, and approximately 10-fold relative to wt (unpublished data). Whether the upregulation of dysferlin and increased MT network stability observed in dKO muscle lead to a more efficient transport of the protein to the sarcolemma and eventually to improved sarcolemma repair, remain interesting questions to be investigated.

Nishimura and colleagues suggested that MTs play an important role in cellular biomechanics [47]. They showed that cardiomyocytes with hyperpolymerized MTs (paclitaxel-treated) exhibit increased shear stiffness compared to untreated cardiomyocytes, whereas in cells with depolymerized MTs (colchicine-treated) a decrease in longitudinal shear stiffness was observed. Visualization of MTs in paclitaxel-treated cardiomyocytes revealed especially the longitudinal MTs to be increased, similar to our observation in skeletal muscle of dKO mice. Therefore, we speculate that mechanical load of dKO myofibers (stiffened through the accumulation of MTs underneath the sarcolemma) could make them more susceptible to bursting (at least relative to wt and cKO myofibers), leading to necrosis that manifests as elevations of CK plasma levels and of neutrophile granulocytes. Increased stiffness would also explain why dKO mice show a more severe structural phenotype compared to cKO and *mdx* mice.

Interestingly, despite exhibiting a reduced number of glycolytic type II fibers, dKO mice do not show diminished glucose uptake. This could be due to MTs lying directly underneath the sarcolemma, thereby positioning GLUT4 vesicles close enough to the membrane to allow glucose uptake even without insulin-triggering. Thus, also at times of low or no food consumption, myofibers are probably competing for glucose and in case of critical shortage, the muscle is switching to oxidative phosphorylation and finally to muscle wasting, leading to smaller fiber diameters, reduced weight, and early death of dKO mice.

Implying an involvement of plectin 1f in metabolic processes, our study adds another important aspect to the functional repertoire of this highly versatile cytolinker protein. Isoform 1f has previously been shown to play a crucial role in muscle maintenance by linking the desmin network to the dystroglycan protein complex [7]. Moreover, it has recently been found that patients bearing mutations in the exon encoding its isoform-specific N-terminal sequence are suffering from limb-girdle muscular dystrophy (mostly affecting shoulder and hip muscles) but not from skin blistering, contrary to patients lacking all isoforms of plectin [48].

In recent years, emphasis has been put on developing genetic and pharmacological therapies for DMD, leading to a deceleration of disease progression. However, a cure for the disease has not become available so far. Previous and present studies have/are focused on either the re-expression of ‘mini-dystrophin’, a shorter version of the protein [49]; or skipping the mutated exon using antisense oligonucleotides [50]; or pharmacologically enabling the ribosome to read through the premature stop codon of the mutated dystrophin gene [51]. None of these approaches led to more than 15% re-expression of dystrophin [52]. This low level of restoration is probably insufficient to overcome the potentially damaging effects, such as deregulation of MT-dependent processes and signal transduction events, caused by plectin’s accumulation at the sarcolemma as seen in DMD patients and *mdx* mice. Thus, for a more efficient rescue of muscular dystrophy in patients, therapeutic strategies aiming at a balanced expression of sarcolemmal levels of both, dystrophin and plectin, could prove useful.

## Conclusions

In conclusion, the comparative phenotypic analyses of mouse lines lacking either dystrophin (*mdx*), or plectin (MCK-Cre/cKO), and of a newly generated double KO mouse line lacking both proteins, revealed that the impairment of glucose uptake observed in *mdx* mice is due to the overexpression of plectin at the sarcolemma of their myofibers. Our results suggest that plectin, upon accumulation at the sarcolemma of *mdx* myofibers, acts as an antagonist of MT network formation thereby impeding MT-dependent delivery of glucose transporter 4 to the membrane. This study adds a novel facet to plectin’s already vast repertoire of functions in cytoskeleton organization and dynamics and it opens an interesting new perspective on mechanisms linking the metabolism of skeletal muscle fibers to their extra-sarcomeric cytoarchitecture. Our study thus provides not only important new insights into pathomechanisms of plectinopathies but also muscular dystrophies in general.

## Abbreviations

CK: Creatine kinase; cKO: Conditional knockout; COX: Cytochrome-c oxidase; dKO: Double knockout; DMD: Duchenne muscular dystrophy; EBD: Evans Blue dye; EBS: Epidermolysis bullosa simplex; EDL: Extensor digitorum longus; GC: Gastrocnemius; GLUT4: Glucose transporter 4; HPRT1: Hypoxanthine guanine phosphoribosyl transferase 1; MAP: Microtubule associated protein; Mdx: Muscular dystrophy X-linked; MT: Microtubule; oGTT: Oral glucose tolerance test; P1: Plectin isoform 1; P1b: Plectin isoform 1b; P1d: Plectin isoform 1d; P1f: Plectin isoform 1f; PAS: Periodic acid-Schiff reaction; PFA: Paraformaldehyde; QF: Quadriceps femoris; SDH: Succinyl dehydrogenase; TG: Trichrome Gomori; Wt: Wild-type.

## Competing interests

The authors declare that they have no competing interests.

## Authors’ contributions

MR, GAR, and GW designed research, MR, RGV, IF, and MO performed experiments, MR, JP, SS, GAR, and GW analyzed data, and MR, GAR, and GW wrote the paper. All authors read and approved the final manuscript.

## Supplementary Material

Additional file 1: Figure S1 Immunoblotting and corresponding Coomassie-stained gel of GC muscle lysates using antibodies to plectin and dystrophin. Note absence of signals for plectin in cKO, for dystrophin in *mdx*, and for both proteins in dKO samples. Positions of molecular mass markers (kDa) are indicated in immunoblots and corresponding Coomassie-stained gel. Click here for file

Additional file 2: Table S1 Summary of phenotypes observed in cKO, *mdx*, and dKO in comparison to wt mice (all values represent mean ± SEM). Click here for file

Additional file 3: Figure S2 Histopathology of soleus and EDL muscles. Haematoxylin & eosin (H&E), cytochrome-c oxidase (COX), succinyl dehydrogenase (SDH), nicotinamide adenine dinucleotide (NADH), periodic acid-Schiff reaction (PAS), trichrome Gomori (TG), and adenosine triphosphatase (ATPase; pH 4.2) stainings of cryosections from 10-week-old wt and mutant mice are shown. Click here for file

Additional file 4: Figure S3 White blood cell count. Bar graph shows white blood cell counts of May-Gruenwald-Giemsa stained blood smears from wt and mutant mice. Note increased number of granulocytes and relative reduction of lymphocytes (characteristics of severe tissue necrosis) in dKO mice (*P < 0.05; n = 5 per genotype; data presented as mean ± SEM). Click here for file

Additional file 5: Figure S4 Control experiment for GLUT4 translocation assays (see Figures 1D and E) using pmCherry-HA (without GLUT4-encoding sequence) instead of pmCherry-HA-GLUT4 for transfection. Note that the expressed mCherry-HA fusion protein is not immunodetectable (not surface-exposed) under nonpermeabilizing conditions, but only after permeabilization of cells. Bar, 10 μm. Click here for file

Additional file 6: Figure S5 Immunofluorescence microscopy of sarcomeric α-actinin in myoblasts used for the quantification of GLUT4 translocation (differentiated for seven days). Note pronounced striated staining pattern, a characteristic feature of mature myofibers. Click here for file

Additional file 7: Figure S6 Micrographs showing representative immunofluorescence images used for MT length assessment (total length of MTs per cell area) in nocodazole-treated primary myofibers. Arrowheads indicate the beginning and end of one of at least 65 MTs measured in each case. Note generally reduced lengths of MTs in *mdx* specimens. Bar, 10 μm. Click here for file
