# Supplementary figures and images for: Linking cytoarchitecture to metabolism: sarcolemma-associated plectin affects glucose uptake by destabilizing microtubule networks in mdx myofibers
Source: Skelet Muscle. 2013 Jun 12;3:14. doi: 10.1186/2044-5040-3-14 (PMC3695810; doi:10.1186/2044-5040-3-14)

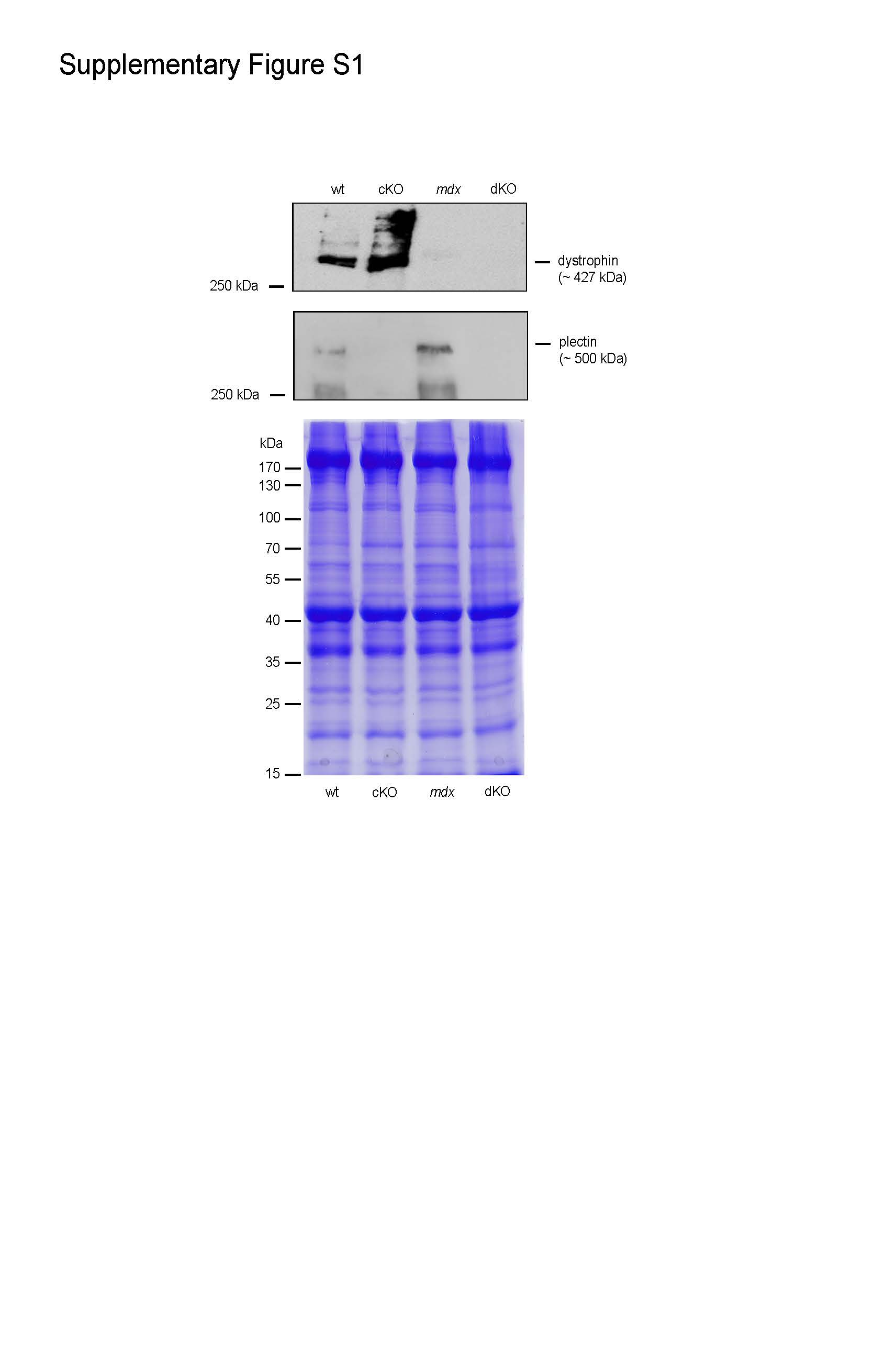

Supplement: Additional file 1: Figure S1 — Immunoblotting and corresponding Coomassie-stained gel of GC muscle lysates using antibodies to plectin and dystrophin. Note absence of signals for plectin in cKO, for dystrophin in mdx, and for both proteins in dKO samples. Positions of molecular mass markers (kDa) are indicated in immunoblots and corresponding Coomassie-stained gel. [file 2044-5040-3-14-S1.jpeg]

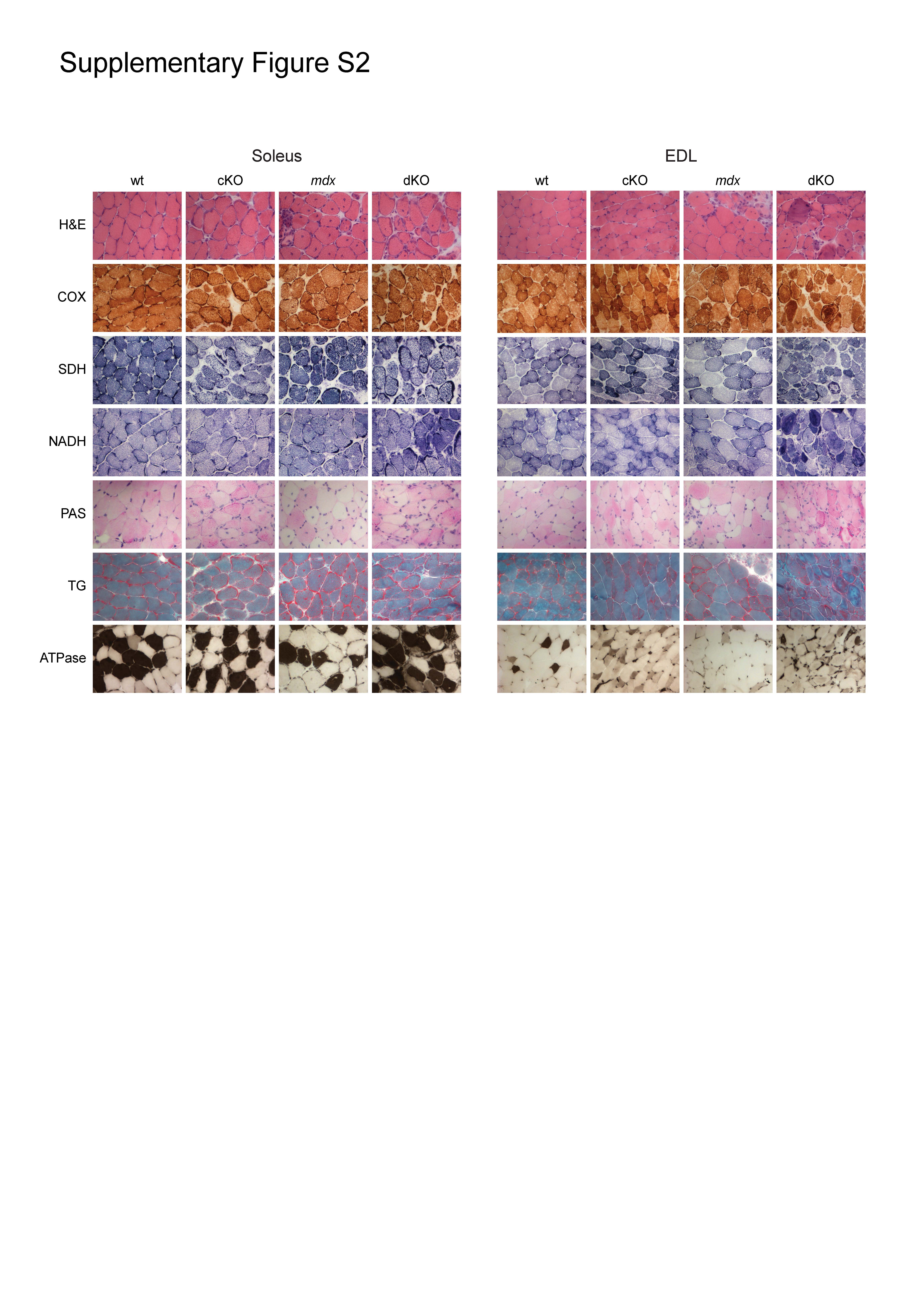

Supplement: Additional file 3: Figure S2 — Histopathology of soleus and EDL muscles. Haematoxylin & eosin (H&E), cytochrome-c oxidase (COX), succinyl dehydrogenase (SDH), nicotinamide adenine dinucleotide (NADH), periodic acid-Schiff reaction (PAS), trichrome Gomori (TG), and adenosine triphosphatase (ATPase; pH 4.2) stainings of cryosections from 10-week-old wt and mutant mice are shown. [file 2044-5040-3-14-S3.jpeg]

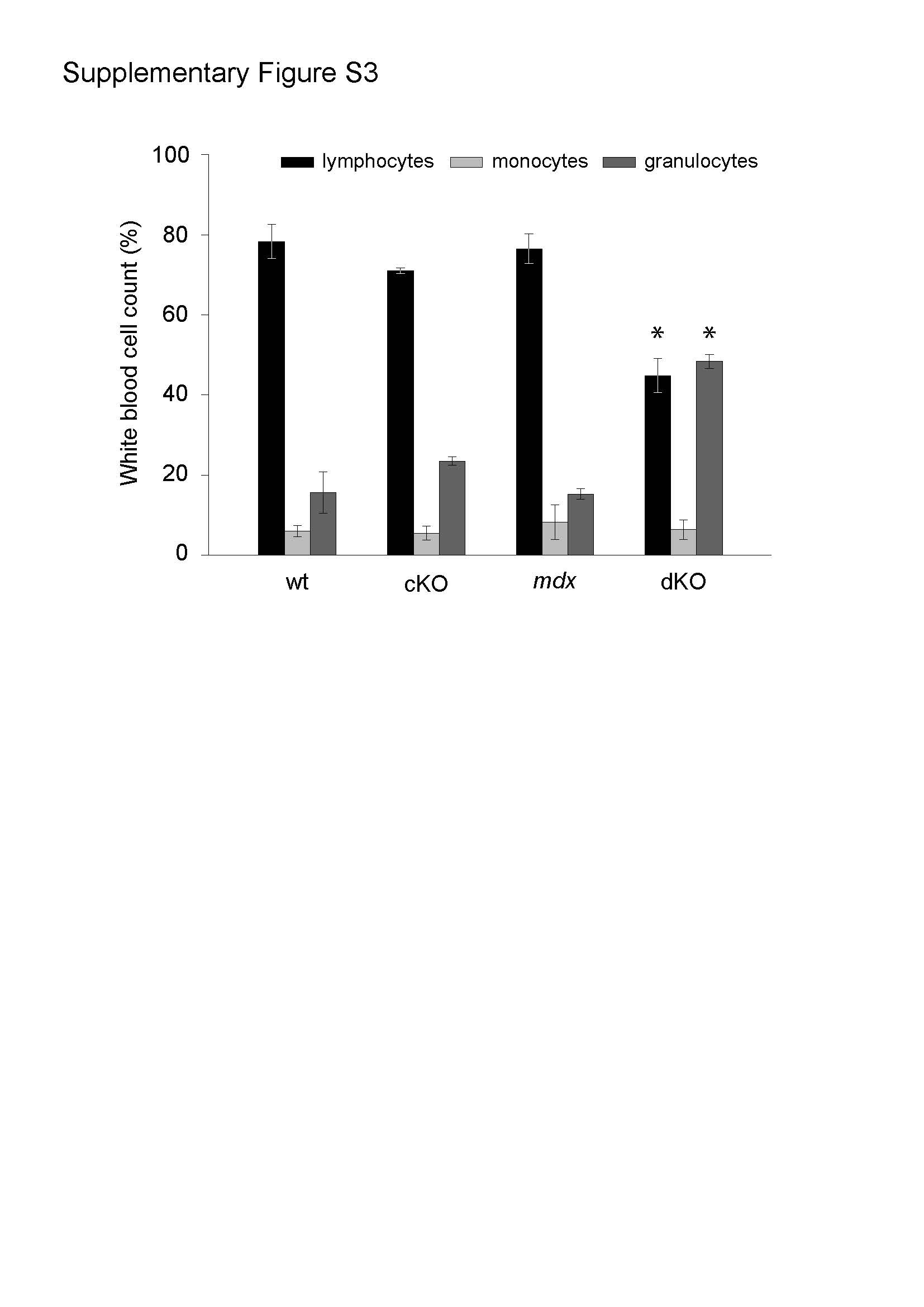

Supplement: Additional file 4: Figure S3 — White blood cell count. Bar graph shows white blood cell counts of May-Gruenwald-Giemsa stained blood smears from wt and mutant mice. Note increased number of granulocytes and relative reduction of lymphocytes (characteristics of severe tissue necrosis) in dKO mice (*P < 0.05; n = 5 per genotype; data presented as mean ± SEM). [file 2044-5040-3-14-S4.jpeg]

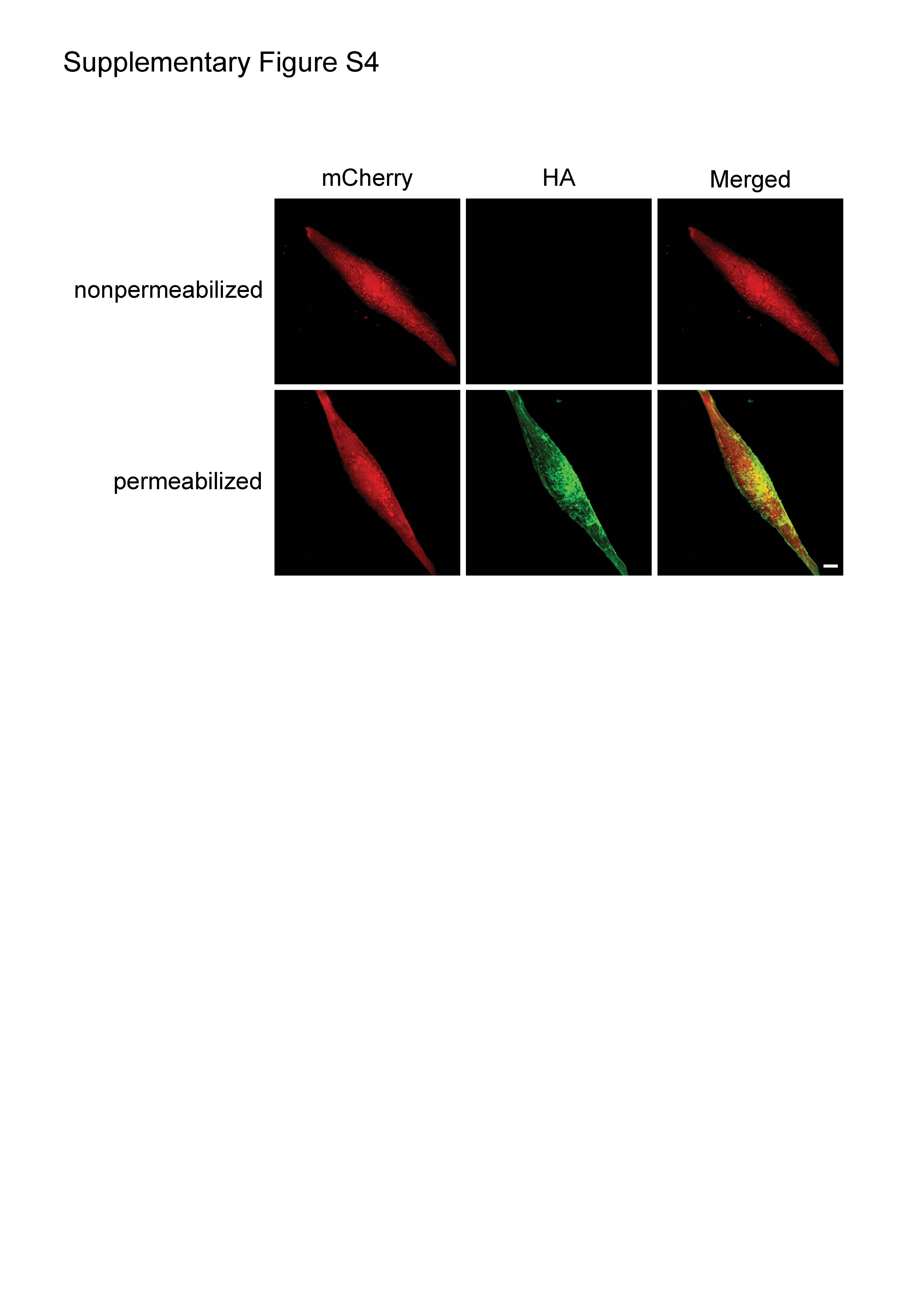

Supplement: Additional file 5: Figure S4 — Control experiment for GLUT4 translocation assays (see Figures 1D and E) using pmCherry-HA (without GLUT4-encoding sequence) instead of pmCherry-HA-GLUT4 for transfection. Note that the expressed mCherry-HA fusion protein is not immunodetectable (not surface-exposed) under nonpermeabilizing conditions, but only after permeabilization of cells. Bar, 10 μm. [file 2044-5040-3-14-S5.jpeg]

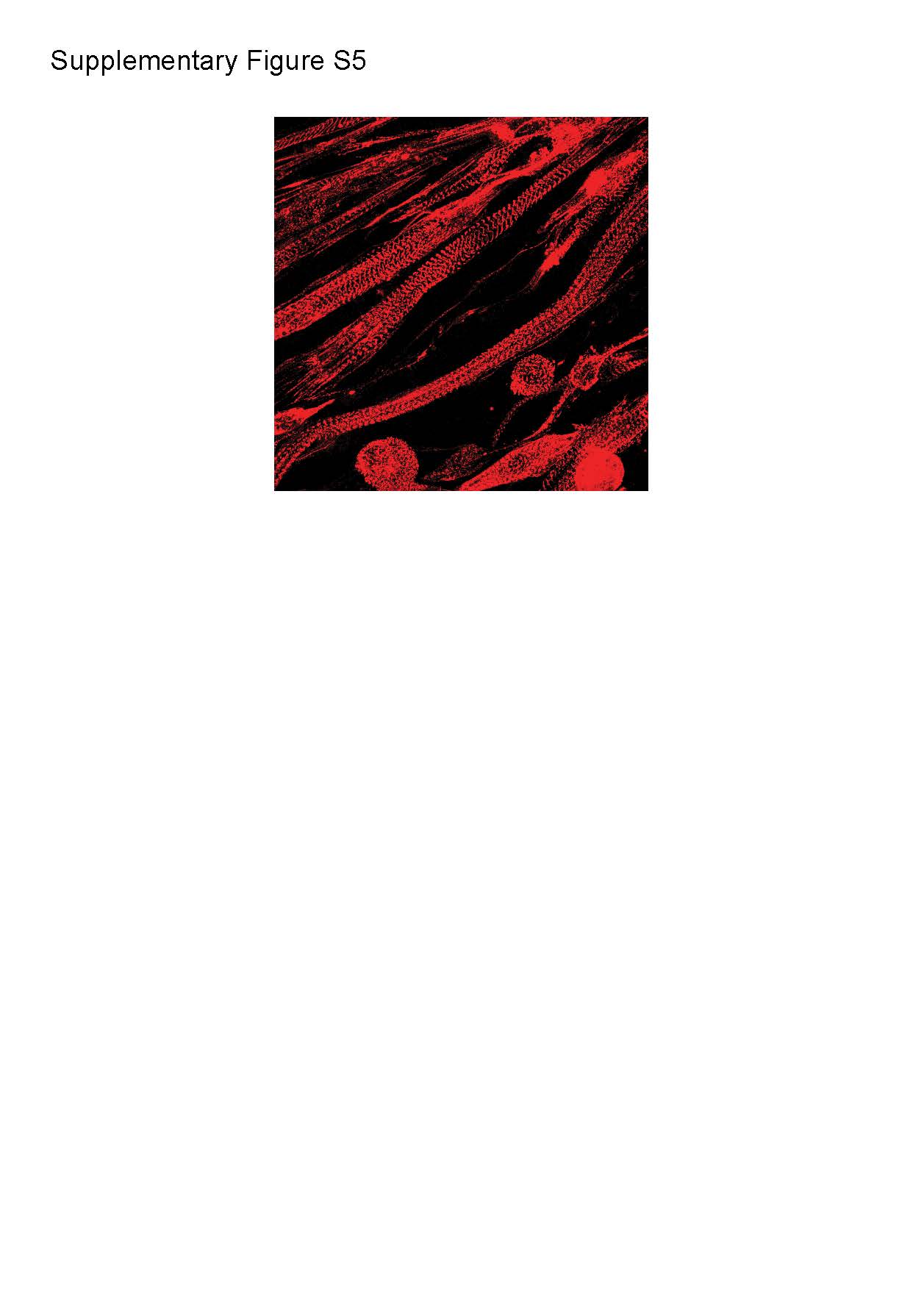

Supplement: Additional file 6: Figure S5 — Immunofluorescence microscopy of sarcomeric α-actinin in myoblasts used for the quantification of GLUT4 translocation (differentiated for seven days). Note pronounced striated staining pattern, a characteristic feature of mature myofibers. [file 2044-5040-3-14-S6.jpeg]

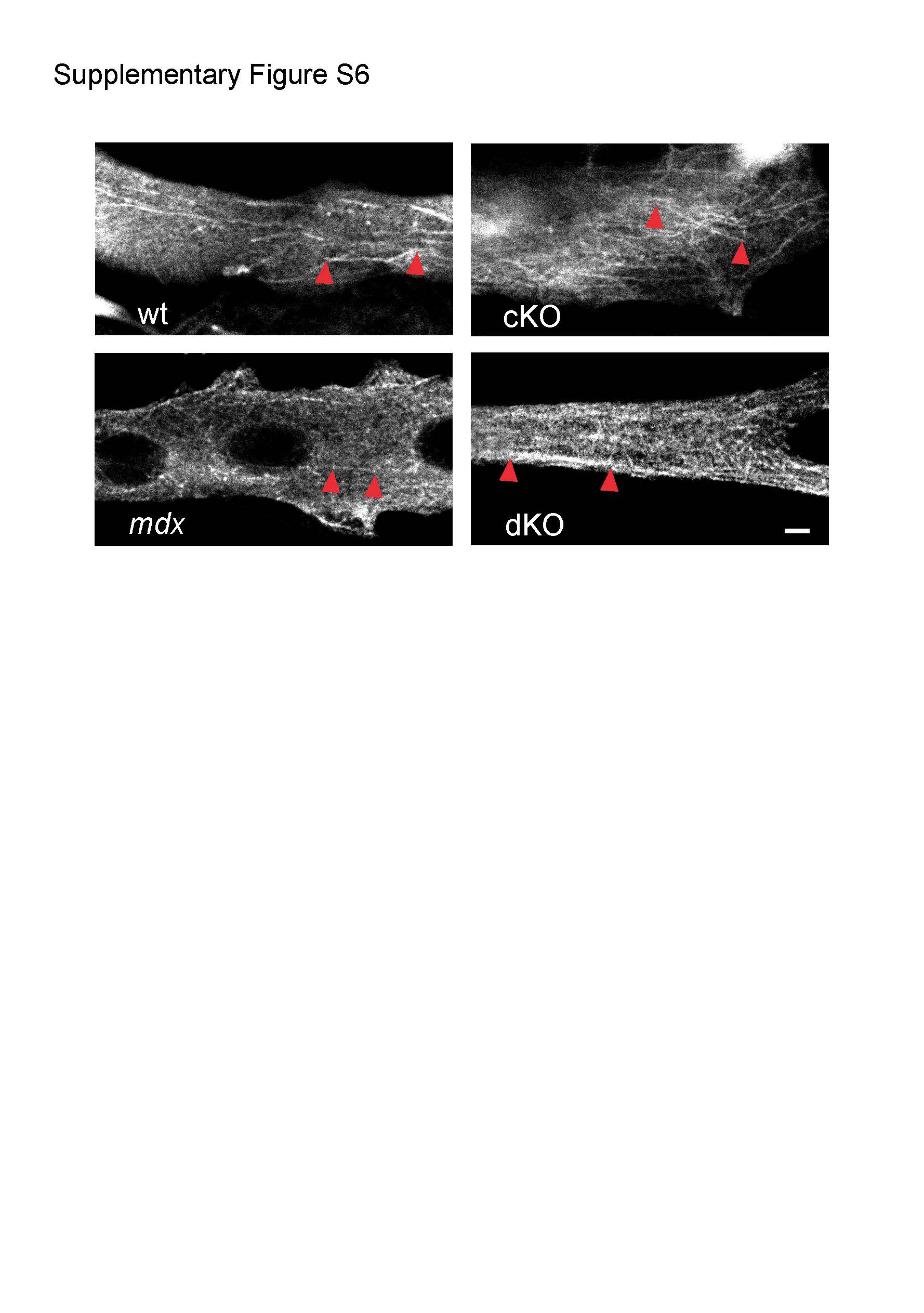

Supplement: Additional file 7: Figure S6 — Micrographs showing representative immunofluorescence images used for MT length assessment (total length of MTs per cell area) in nocodazole-treated primary myofibers. Arrowheads indicate the beginning and end of one of at least 65 MTs measured in each case. Note generally reduced lengths of MTs in mdx specimens. Bar, 10 μm. [file 2044-5040-3-14-S7.jpeg]
