# Supplementary material for: Linking cytoarchitecture to metabolism: sarcolemma-associated plectin affects glucose uptake by destabilizing microtubule networks in mdx myofibers
Source: Skelet Muscle. 2013 Jun 12;3:14. doi: 10.1186/2044-5040-3-14 (PMC3695810; doi:10.1186/2044-5040-3-14)
Supplement: Additional file 2: Table S1 — Summary of phenotypes observed in cKO, mdx, and dKO in comparison to wt mice (all values represent mean ± SEM). [file 2044-5040-3-14-S2.doc]

**Supplementary Table 1.** Summary of phenotypes observed in cKO, *mdx*, and dKO in com­pari­son to wt mice (all values represent mean ± SEM).

|  | **Wt** | **cKO** | ***mdx*** | **dKO** |
| --- | --- | --- | --- | --- |
| **Weight** | normal | normal | increased | reduced |
| **H&E** | normal | few regenerated and atrophic | fibrosis | highly atrophic and fibrotic |
| **Central nuclei** | none | few | many | few |
| **COX** | normal | **slightly pathologic** | decreased at fiber center, increased at the sarcolemma | decreased at fiber center, increased at the sarcolemma |
| **SDH** | normal | **slightly pathologic** | decreased at fiber center, increased at the sarcolemma | decreased at fiber center, increased at the sarcolemma |
| **NADH** | normal | normal | normal | strongly positive |
| **PAS** | normal | normal | decreased | normal |
| **Trichrome Gomori** | normal | mitochondrial aggregates | normal | mitochondrial aggregates |
| **ATPase** | normal | normal | normal | type II fibers reduced |
| **Fiber diameters** | normal | type II fibers with smaller diameters | high number of hypertrophic myofibers | normal |
| **Differential blood count** | normal | normal | normal | increase of neutrophile granulocytes |
| **Life expectancy** | normal | > 6 months | 21 months | 5-17 weeks |
| **EBD positive fibers** | 0 | 0 | 13.5 ± 0.4 | 0 |
| **CK activity in plasma (U/L)** | 89 ± 11 | 85 ± 5 | 2,651 ± 493 | 685 ± 83 |
| **CK activity in muscle lysates (10^5^ U/l)** | 14.48 ± 41.40 | 3.99 ± 0.75 | 2.68 ± 0.29 | 5.60 ± 1.03 |
| **CK mRNA levels** | 1.00 ± 0.23 | 0.40 ± 0.06 | 0.33 ± 0.05 | 0.23 ± 0.03 |
| **oGTT** | normal | normal | increased at 45 and 60 minutes | normal |
| **Insulin levels** | normal | normal | increased due to higher glucose levels | normal |
| **GLUT4 protein levels (%)** | 100 ± | 106 ± 8 | 90 ± 16 | 120 ± 12 |
| **α-Tubulin staining of TMF** | normal | prominent, disorganized MT network | reduced at the sarcolemma, disorganized MT network | thick, longitudinal bundles of MT underneath the sarcolemma |
| **α-Tubulin protein levels (%)** | 100 ± 31 | 480 ± 104 | 699 ± 139 | 808 ± 204 |
| **Acetylated tubulin levels (%)** | 100 ± 25 | 106 ± 20 | 132 ± 21 | 104 ± 18 |
| **Length of MTs after nocodazole treatment normalized to cell area** |  | 6.65e-3 ± 1.14e-3 | 4.23e-3 ± 2.74e-4 | 5.93e-3 ± 7.27e-4 |
| **HMW tau protein levels (%)** | 100 ± 11 | 164 ± 10 | 110 ± 5 | 174 ± 19 |
